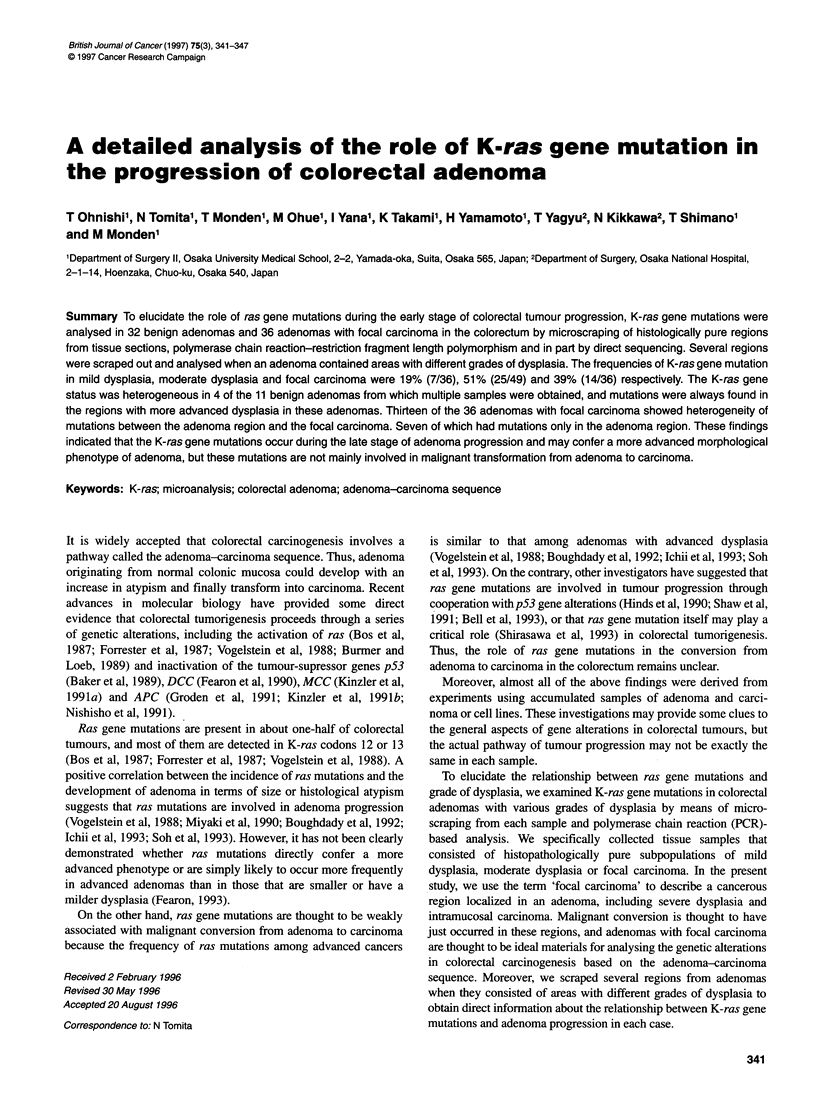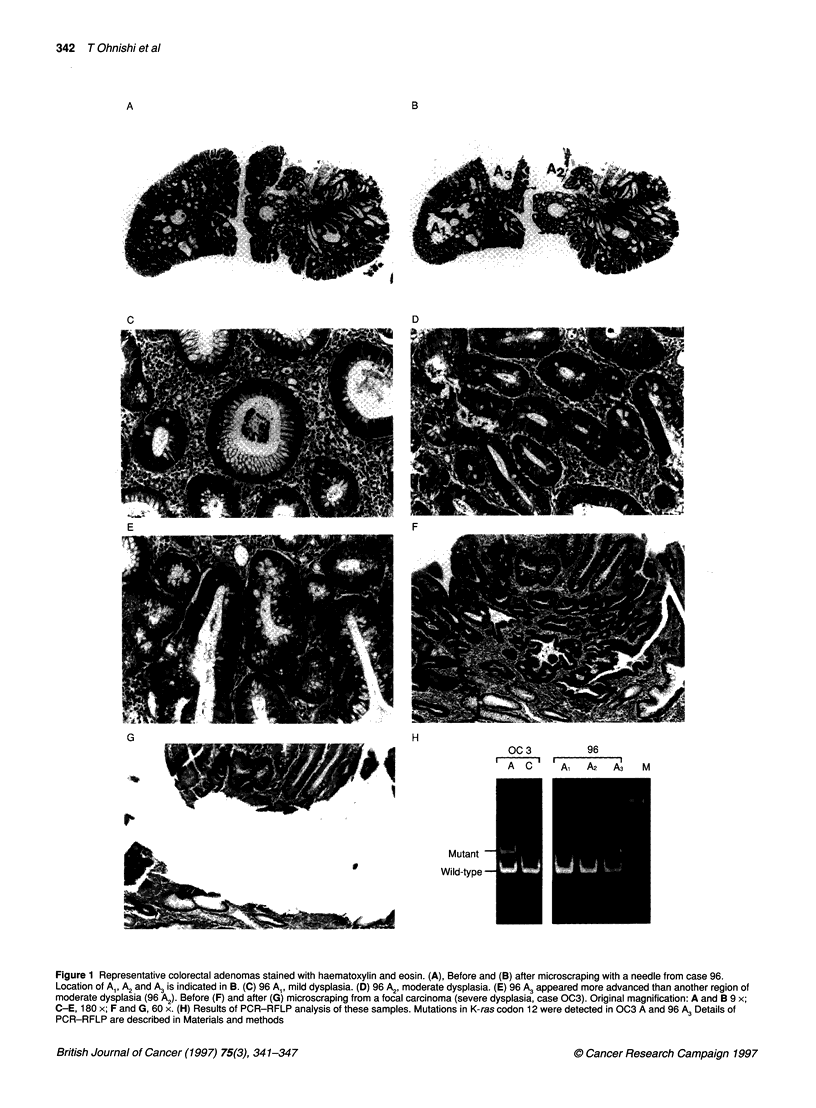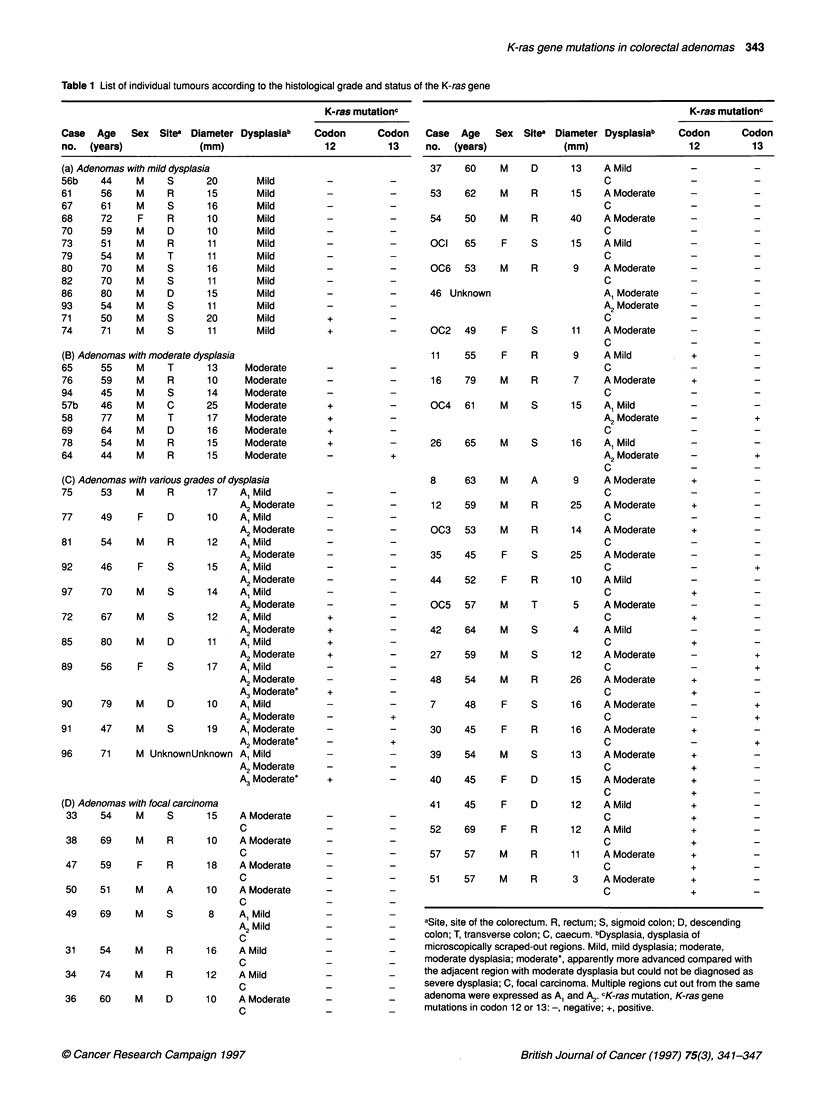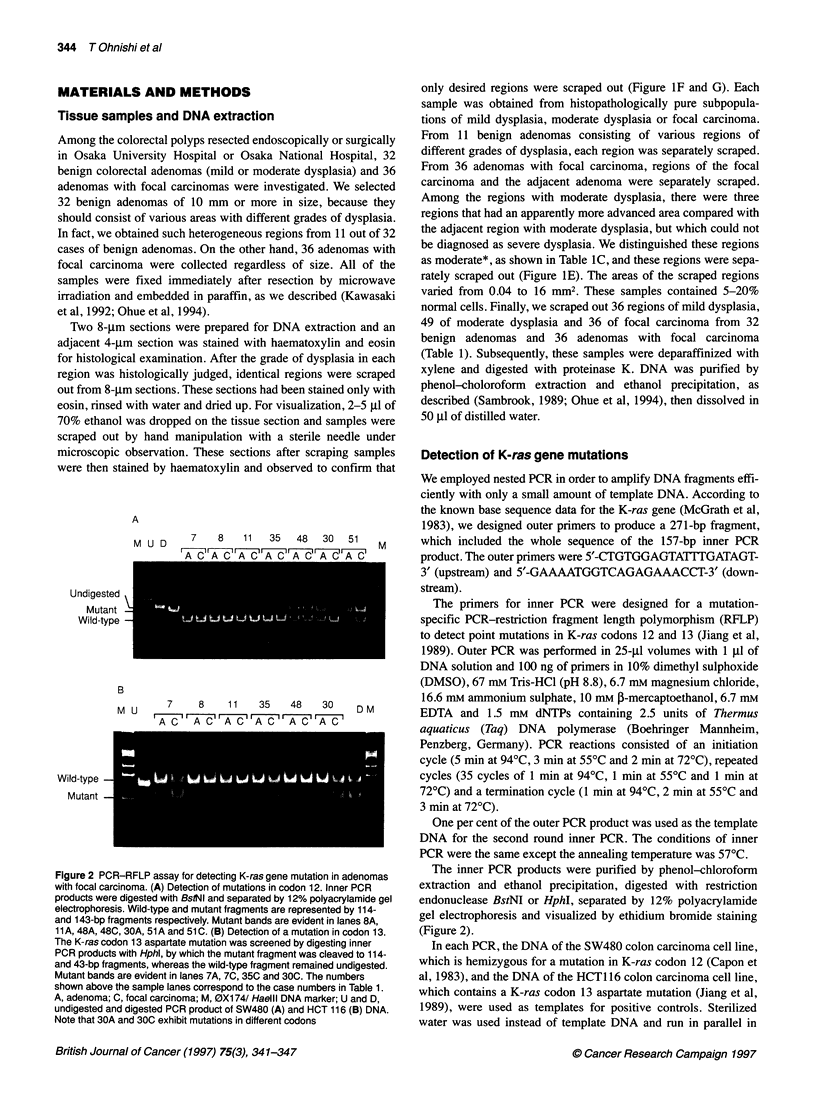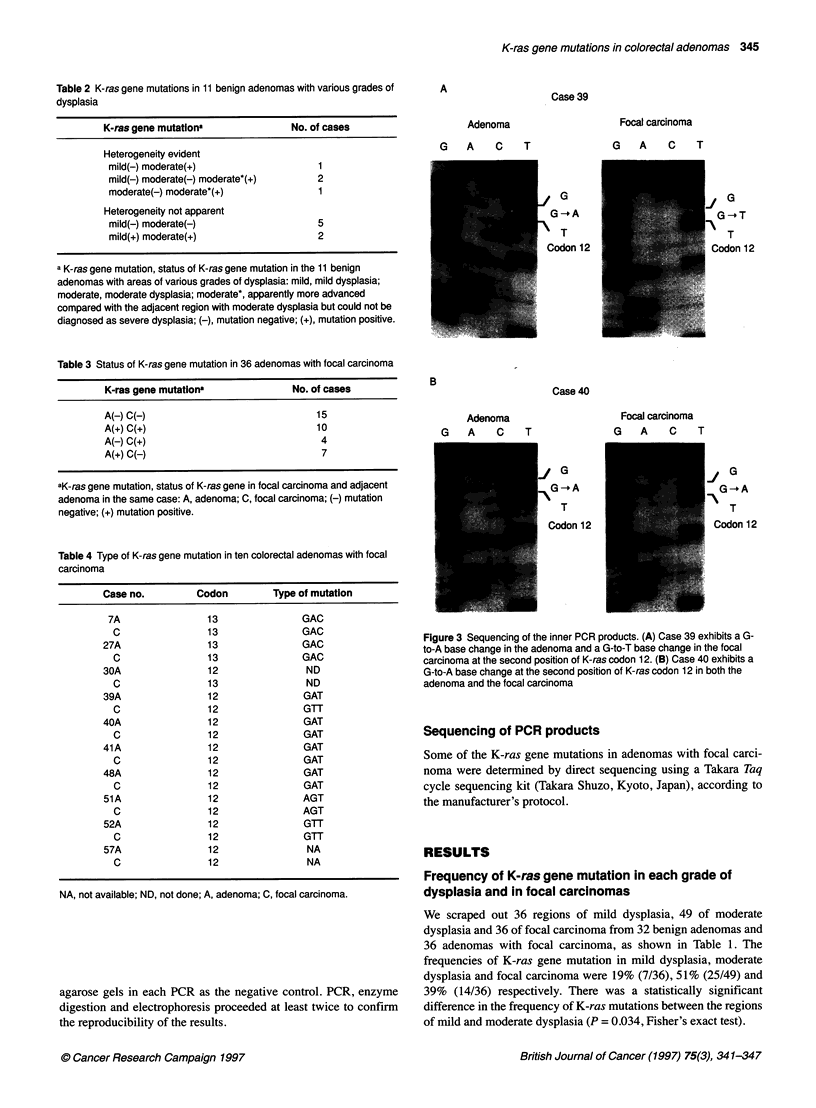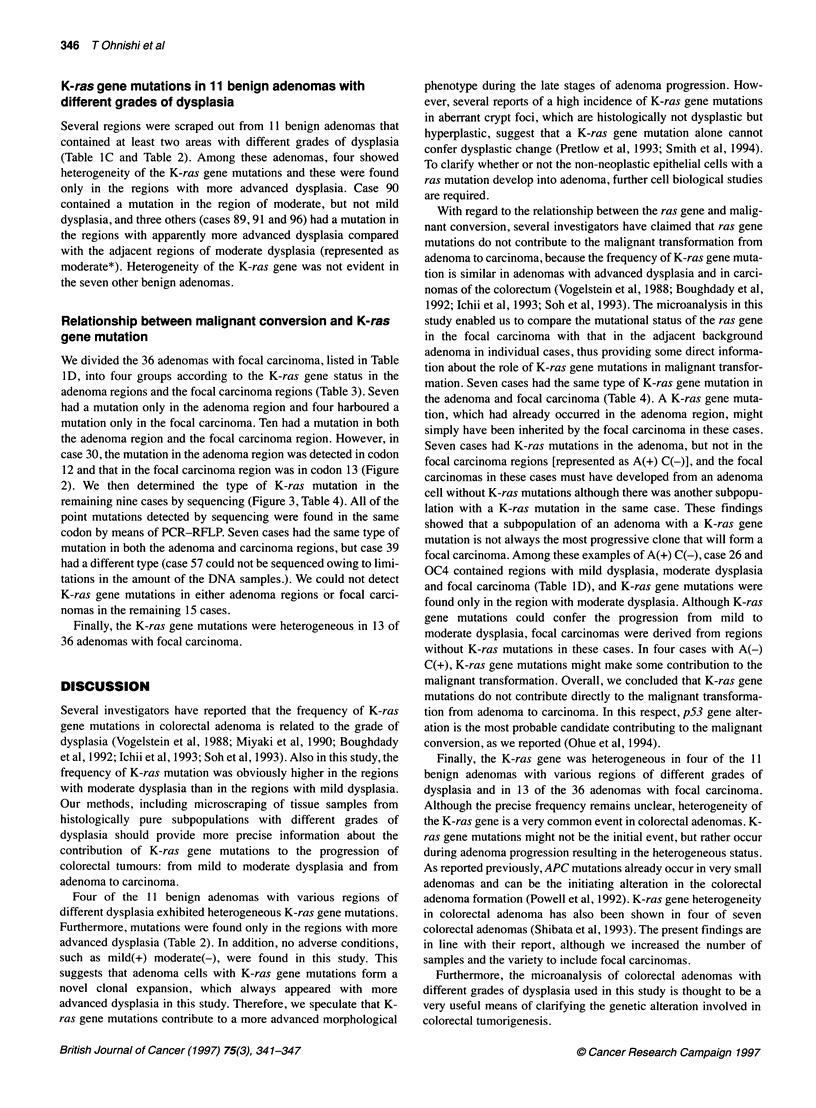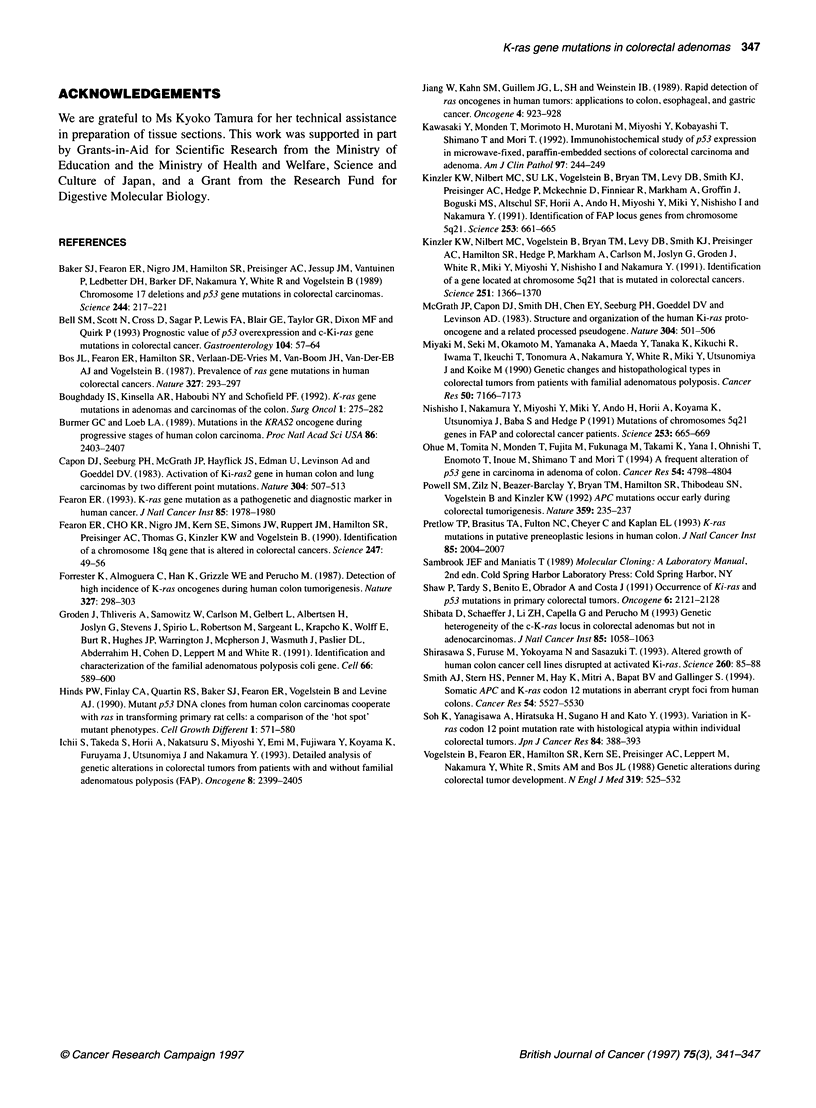# A detailed analysis of the role of K-ras gene mutation in the progression of colorectal adenoma.

**DOI:** 10.1038/bjc.1997.56

**Published:** 1997

**Authors:** T. Ohnishi, N. Tomita, T. Monden, M. Ohue, I. Yana, K. Takami, H. Yamamoto, T. Yagyu, N. Kikkawa, T. Shimano, M. Monden

**Affiliations:** Department of Surgery II, Osaka University Medical School, Yamada-oka, Suita, Japan.

## Abstract

**Images:**


*British Journal of Cancer* (1997) **75**(3), 341–347
© 1997 Cancer Research Campaign

### A detailed analysis of the role of K-*ras* gene mutation in the progression of colorectal adenoma

**T Ohnishi[1], N Tomita[1], T Monden[1], M Ohue[1], I Yana[1], K Takami[1], H Yamamoto[1], T Yagyu[2], N Kikkawa[2], T Shimano[1] and M Monden[1]**

[1]Department of Surgery II, Osaka University Medical School, 2–2, Yamada-oka, Suita, Osaka 565, Japan; [2]Department of Surgery, Osaka National Hospital, 2–1–14, Hoenzaka, Chuo-ku, Osaka 540, Japan

**Summary** To elucidate the role of *ras* gene mutations during the early stage of colorectal tumour progression, K-*ras* gene mutations were analysed in 32 benign adenomas and 36 adenomas with focal carcinoma in the colorectum by microscraping of histologically pure regions from tissue sections, polymerase chain reaction–restriction fragment length polymorphism and in part by direct sequencing. Several regions were scraped out and analysed when an adenoma contained areas with different grades of dysplasia. The frequencies of K-*ras* gene mutation in mild dysplasia, moderate dysplasia and focal carcinoma were 19% (7/36), 51% (25/49) and 39% (14/36) respectively. The K-*ras* gene status was heterogeneous in 4 of the 11 benign adenomas from which multiple samples were obtained, and mutations were always found in the regions with more advanced dysplasia in these adenomas. Thirteen of the 36 adenomas with focal carcinoma showed heterogeneity of mutations between the adenoma region and the focal carcinoma. Seven of which had mutations only in the adenoma region. These findings indicated that the K-*ras* gene mutations occur during the late stage of adenoma progression and may confer a more advanced morphological phenotype of adenoma, but these mutations are not mainly involved in malignant transformation from adenoma to carcinoma.

**Keywords:** K-*ras*; microanalysis; colorectal adenoma; adenoma–carcinoma sequence

It is widely accepted that colorectal carcinogenesis involves a pathway called the adenoma–carcinoma sequence. Thus, adenoma originating from normal colonic mucosa could develop with an increase in atypism and finally transform into carcinoma. Recent advances in molecular biology have provided some direct evidence that colorectal tumorigenesis proceeds through a series of genetic alterations, including the activation of *ras* (Bos et al, 1987; Forrester et al, 1987; Vogelstein et al, 1988; Burmer and Loeb, 1989) and inactivation of the tumour-supressor genes *p53* (Baker et al, 1989), *DCC* (Fearon et al, 1990), *MCC* (Kinzler et al, 1991*a*) and *APC* (Groden et al, 1991; Kinzler et al, 1991*b*; Nishisho et al, 1991).

*Ras* gene mutations are present in about one-half of colorectal tumours, and most of them are detected in K-*ras* codons 12 or 13 (Bos et al, 1987; Forrester et al, 1987; Vogelstein et al, 1988). A positive correlation between the incidence of *ras* mutations and the development of adenoma in terms of size or histological atypism suggests that *ras* mutations are involved in adenoma progression (Vogelstein et al, 1988; Miyaki et al, 1990; Boughdady et al, 1992; Ichii et al, 1993; Soh et al, 1993). However, it has not been clearly demonstrated whether *ras* mutations directly confer a more advanced phenotype or are simply likely to occur more frequently in advanced adenomas than in those that are smaller or have a milder dysplasia (Fearon, 1993).

On the other hand, *ras* gene mutations are thought to be weakly associated with malignant conversion from adenoma to carcinoma because the frequency of *ras* mutations among advanced cancers is similar to that among adenomas with advanced dysplasia (Vogelstein et al, 1988; Boughdady et al, 1992; Ichii et al, 1993; Soh et al, 1993). On the contrary, other investigators have suggested that *ras* gene mutations are involved in tumour progression through cooperation with *p53* gene alterations (Hinds et al, 1990; Shaw et al, 1991; Bell et al, 1993), or that *ras* gene mutation itself may play a critical role (Shirasawa et al, 1993) in colorectal tumorigenesis. Thus, the role of *ras* gene mutations in the conversion from adenoma to carcinoma in the colorectum remains unclear.

Moreover, almost all of the above findings were derived from experiments using accumulated samples of adenoma and carcinoma or cell lines. These investigations may provide some clues to the general aspects of gene alterations in colorectal tumours, but the actual pathway of tumour progression may not be exactly the same in each sample.

To elucidate the relationship between *ras* gene mutations and grade of dysplasia, we examined K-*ras* gene mutations in colorectal adenomas with various grades of dysplasia by means of microscraping from each sample and polymerase chain reaction (PCR)-based analysis. We specifically collected tissue samples that consisted of histopathologically pure subpopulations of mild dysplasia, moderate dysplasia or focal carcinoma. In the present study, we use the term 'focal carcinoma' to describe a cancerous region localized in an adenoma, including severe dysplasia and intramucosal carcinoma. Malignant conversion is thought to have just occurred in these regions, and adenomas with focal carcinoma are thought to be ideal materials for analysing the genetic alterations in colorectal carcinogenesis based on the adenoma–carcinoma sequence. Moreover, we scraped several regions from adenomas when they consisted of areas with different grades of dysplasia to obtain direct information about the relationship between K-*ras* gene mutations and adenoma progression in each case.

*Received 2 February 1996*
*Revised 30 May 1996*
*Accepted 20 August 1996*

*Correspondence to:* N Tomita

**Figure 1** Representative colorectal adenomas stained with haematoxylin and eosin. (**A**), Before and (**B**) after microscraping with a needle from case 96. Location of $A_1$, $A_2$ and $A_3$ is indicated in **B**. (**C**) 96 $A_1$, mild dysplasia. (**D**) 96 $A_2$, moderate dysplasia. (**E**) 96 $A_3$ appeared more advanced than another region of moderate dysplasia (96 $A_2$). Before (**F**) and after (**G**) microscraping from a focal carcinoma (severe dysplasia, case OC3). Original magnification: **A** and **B** 9 ×; **C–E**, 180 ×; **F** and **G**, 60 ×. (**H**) Results of PCR–RFLP analysis of these samples. Mutations in K-*ras* codon 12 were detected in OC3 A and 96 $A_3$ Details of PCR–RFLP are described in Materials and methods

© Cancer Research Campaign 1997

**Table 1** List of individual tumours according to the histological grade and status of the K-*ras* gene

| Case no. | Age (years) | Sex | Site[a] | Diameter (mm) | Dysplasia[b] | K-*ras* mutation[c] Codon 12 | K-*ras* mutation[c] Codon 13 |
|---|---|---|---|---|---|---|---|
| (a) *Adenomas with mild dysplasia* | | | | | | | |
| 56b | 44 | M | S | 20 | Mild | – | – |
| 61 | 56 | M | R | 15 | Mild | – | – |
| 67 | 61 | M | S | 16 | Mild | – | – |
| 68 | 72 | F | R | 10 | Mild | – | – |
| 70 | 59 | M | D | 10 | Mild | – | – |
| 73 | 51 | M | R | 11 | Mild | – | – |
| 79 | 54 | M | T | 11 | Mild | – | – |
| 80 | 70 | M | S | 16 | Mild | – | – |
| 82 | 70 | M | S | 11 | Mild | – | – |
| 86 | 80 | M | D | 15 | Mild | – | – |
| 93 | 54 | M | S | 11 | Mild | – | – |
| 71 | 50 | M | S | 20 | Mild | + | – |
| 74 | 71 | M | S | 11 | Mild | + | – |
| (B) *Adenomas with moderate dysplasia* | | | | | | | |
| 65 | 55 | M | T | 13 | Moderate | – | – |
| 76 | 59 | M | R | 10 | Moderate | – | – |
| 94 | 45 | M | S | 14 | Moderate | – | – |
| 57b | 46 | M | C | 25 | Moderate | + | – |
| 58 | 77 | M | T | 17 | Moderate | + | – |
| 69 | 64 | M | D | 16 | Moderate | + | – |
| 78 | 54 | M | R | 15 | Moderate | + | – |
| 64 | 44 | M | R | 15 | Moderate | – | + |
| (C) *Adenomas with various grades of dysplasia* | | | | | | | |
| 75 | 53 | M | R | 17 | $A_1$ Mild | – | – |
| | | | | | $A_2$ Moderate | – | – |
| 77 | 49 | F | D | 10 | $A_1$ Mild | – | – |
| | | | | | $A_2$ Moderate | – | – |
| 81 | 54 | M | R | 12 | $A_1$ Mild | – | – |
| | | | | | $A_2$ Moderate | – | – |
| 92 | 46 | F | S | 15 | $A_1$ Mild | – | – |
| | | | | | $A_2$ Moderate | – | – |
| 97 | 70 | M | S | 14 | $A_1$ Mild | – | – |
| | | | | | $A_2$ Moderate | – | – |
| 72 | 67 | M | S | 12 | $A_1$ Mild | + | – |
| | | | | | $A_2$ Moderate | + | – |
| 85 | 80 | M | D | 11 | $A_1$ Mild | + | – |
| | | | | | $A_2$ Moderate | + | – |
| 89 | 56 | F | S | 17 | $A_1$ Mild | – | – |
| | | | | | $A_2$ Moderate | – | – |
| | | | | | $A_3$ Moderate* | + | – |
| 90 | 79 | M | D | 10 | $A_1$ Mild | – | – |
| | | | | | $A_2$ Moderate | – | + |
| 91 | 47 | M | S | 19 | $A_1$ Moderate | – | – |
| | | | | | $A_2$ Moderate* | – | + |
| 96 | 71 | M | Unknown | Unknown | $A_1$ Mild | – | – |
| | | | | | $A_2$ Moderate | – | – |
| | | | | | $A_3$ Moderate* | + | – |
| (D) *Adenomas with focal carcinoma* | | | | | | | |
| 33 | 54 | M | S | 15 | A Moderate | – | – |
| | | | | | C | – | – |
| 38 | 69 | M | R | 10 | A Moderate | – | – |
| | | | | | C | – | – |
| 47 | 59 | F | R | 18 | A Moderate | – | – |
| | | | | | C | – | – |
| 50 | 51 | M | A | 10 | A Moderate | – | – |
| | | | | | C | – | – |
| 49 | 69 | M | S | 8 | $A_1$ Mild | – | – |
| | | | | | $A_2$ Mild | – | – |
| | | | | | C | – | – |
| 31 | 54 | M | R | 16 | A Mild | – | – |
| | | | | | C | – | – |
| 34 | 74 | M | R | 12 | A Mild | – | – |
| | | | | | C | – | – |
| 36 | 60 | M | D | 10 | A Moderate | – | – |
| | | | | | C | – | – |
| 37 | 60 | M | D | 13 | A Mild | – | – |
| | | | | | C | – | – |
| 53 | 62 | M | R | 15 | A Moderate | – | – |
| | | | | | C | – | – |
| 54 | 50 | M | R | 40 | A Moderate | – | – |
| | | | | | C | – | – |
| OCI | 65 | F | S | 15 | A Mild | – | – |
| | | | | | C | – | – |
| OC6 | 53 | M | R | 9 | A Moderate | – | – |
| | | | | | C | – | – |
| 46 | Unknown | | | | $A_1$ Moderate | – | – |
| | | | | | $A_2$ Moderate | – | – |
| | | | | | C | – | – |
| OC2 | 49 | F | S | 11 | A Moderate | – | – |
| | | | | | C | – | – |
| 11 | 55 | F | R | 9 | A Mild | + | – |
| | | | | | C | – | – |
| 16 | 79 | M | R | 7 | A Moderate | + | – |
| | | | | | C | – | – |
| OC4 | 61 | M | S | 15 | $A_1$ Mild | – | – |
| | | | | | $A_2$ Moderate | – | + |
| | | | | | C | – | – |
| 26 | 65 | M | S | 16 | $A_1$ Mild | – | – |
| | | | | | $A_2$ Moderate | – | + |
| | | | | | C | – | – |
| 8 | 63 | M | A | 9 | A Moderate | + | – |
| | | | | | C | – | – |
| 12 | 59 | M | R | 25 | A Moderate | + | – |
| | | | | | C | – | – |
| OC3 | 53 | M | R | 14 | A Moderate | + | – |
| | | | | | C | – | – |
| 35 | 45 | F | S | 25 | A Moderate | – | – |
| | | | | | C | – | + |
| 44 | 52 | F | R | 10 | A Mild | – | – |
| | | | | | C | + | – |
| OC5 | 57 | M | T | 5 | A Moderate | – | – |
| | | | | | C | + | – |
| 42 | 64 | M | S | 4 | A Mild | – | – |
| | | | | | C | + | – |
| 27 | 59 | M | S | 12 | A Moderate | – | + |
| | | | | | C | – | + |
| 48 | 54 | M | R | 26 | A Moderate | + | – |
| | | | | | C | + | – |
| 7 | 48 | F | S | 16 | A Moderate | – | + |
| | | | | | C | – | + |
| 30 | 45 | F | R | 16 | A Moderate | + | – |
| | | | | | C | – | + |
| 39 | 54 | M | S | 13 | A Moderate | + | – |
| | | | | | C | + | – |
| 40 | 45 | F | D | 15 | A Moderate | + | – |
| | | | | | C | + | – |
| 41 | 45 | F | D | 12 | A Mild | + | – |
| | | | | | C | + | – |
| 52 | 69 | F | R | 12 | A Mild | + | – |
| | | | | | C | + | – |
| 57 | 57 | M | R | 11 | A Moderate | + | – |
| | | | | | C | + | – |
| 51 | 57 | M | R | 3 | A Moderate | + | – |
| | | | | | C | + | – |

[a]Site, site of the colorectum. R, rectum; S, sigmoid colon; D, descending colon; T, transverse colon; C, caecum. [b]Dysplasia, dysplasia of microscopically scraped-out regions. Mild, mild dysplasia; moderate, moderate dysplasia; moderate*, apparently more advanced compared with the adjacent region with moderate dysplasia but could not be diagnosed as severe dysplasia; C, focal carcinoma. Multiple regions cut out from the same adenoma were expressed as $A_1$ and $A_2$. [c]*K-ras* mutation, *K-ras* gene mutations in codon 12 or 13: –, negative; +, positive.

### MATERIALS AND METHODS

### Tissue samples and DNA extraction

Among the colorectal polyps resected endoscopically or surgically in Osaka University Hospital or Osaka National Hospital, 32 benign colorectal adenomas (mild or moderate dysplasia) and 36 adenomas with focal carcinomas were investigated. We selected 32 benign adenomas of 10 mm or more in size, because they should consist of various areas with different grades of dysplasia. In fact, we obtained such heterogeneous regions from 11 out of 32 cases of benign adenomas. On the other hand, 36 adenomas with focal carcinoma were collected regardless of size. All of the samples were fixed immediately after resection by microwave irradiation and embedded in paraffin, as we described (Kawasaki et al, 1992; Ohue et al, 1994).

Two 8-µm sections were prepared for DNA extraction and an adjacent 4-µm section was stained with haematoxylin and eosin for histological examination. After the grade of dysplasia in each region was histologically judged, identical regions were scraped out from 8-µm sections. These sections had been stained only with eosin, rinsed with water and dried up. For visualization, 2–5 µl of 70% ethanol was dropped on the tissue section and samples were scraped out by hand manipulation with a sterile needle under microscopic observation. These sections after scraping samples were then stained by haematoxylin and observed to confirm that only desired regions were scraped out (Figure 1F and G). Each sample was obtained from histopathologically pure subpopulations of mild dysplasia, moderate dysplasia or focal carcinoma. From 11 benign adenomas consisting of various regions of different grades of dysplasia, each region was separately scraped. From 36 adenomas with focal carcinoma, regions of the focal carcinoma and the adjacent adenoma were separately scraped. Among the regions with moderate dysplasia, there were three regions that had an apparently more advanced area compared with the adjacent region with moderate dysplasia, but which could not be diagnosed as severe dysplasia. We distinguished these regions as moderate*, as shown in Table 1C, and these regions were separately scraped out (Figure 1E). The areas of the scraped regions varied from 0.04 to 16 mm². These samples contained 5–20% normal cells. Finally, we scraped out 36 regions of mild dysplasia, 49 of moderate dysplasia and 36 of focal carcinoma from 32 benign adenomas and 36 adenomas with focal carcinoma (Table 1). Subsequently, these samples were deparaffinized with xylene and digested with proteinase K. DNA was purified by phenol–choloroform extraction and ethanol precipitation, as described (Sambrook, 1989; Ohue et al, 1994), then dissolved in 50 µl of distilled water.

**Figure 2** PCR–RFLP assay for detecting K-*ras* gene mutation in adenomas with focal carcinoma. (**A**) Detection of mutations in codon 12. Inner PCR products were digested with *Bst*NI and separated by 12% polyacrylamide gel electrophoresis. Wild-type and mutant fragments are represented by 114- and 143-bp fragments respectively. Mutant bands are evident in lanes 8A, 11A, 48A, 48C, 30A, 51A and 51C. (**B**) Detection of a mutation in codon 13. The K-*ras* codon 13 aspartate mutation was screened by digesting inner PCR products with *Hph*I, by which the mutant fragment was cleaved to 114- and 43-bp fragments, whereas the wild-type fragment remained undigested. Mutant bands are evident in lanes 7A, 7C, 35C and 30C. The numbers shown above the sample lanes correspond to the case numbers in Table 1. A, adenoma; C, focal carcinoma; M, ØX174/ *Hae*III DNA marker; U and D, undigested and digested PCR product of SW480 (**A**) and HCT 116 (**B**) DNA. Note that 30A and 30C exhibit mutations in different codons

### Detection of K-*ras* gene mutations

We employed nested PCR in order to amplify DNA fragments efficiently with only a small amount of template DNA. According to the known base sequence data for the K-*ras* gene (McGrath et al, 1983), we designed outer primers to produce a 271-bp fragment, which included the whole sequence of the 157-bp inner PCR product. The outer primers were 5′-CTGTGGAGTATTTGATAGT-3′ (upstream) and 5′-GAAAATGGTCAGAGAAACCT-3′ (downstream).

The primers for inner PCR were designed for a mutation-specific PCR–restriction fragment length polymorphism (RFLP) to detect point mutations in K-*ras* codons 12 and 13 (Jiang et al, 1989). Outer PCR was performed in 25-µl volumes with 1 µl of DNA solution and 100 ng of primers in 10% dimethyl sulphoxide (DMSO), 67 mM Tris-HCl (pH 8.8), 6.7 mM magnesium chloride, 16.6 mM ammonium sulphate, 10 mM β-mercaptoethanol, 6.7 mM EDTA and 1.5 mM dNTPs containing 2.5 units of *Thermus aquaticus* (*Taq*) DNA polymerase (Boehringer Mannheim, Penzberg, Germany). PCR reactions consisted of an initiation cycle (5 min at 94°C, 3 min at 55°C and 2 min at 72°C), repeated cycles (35 cycles of 1 min at 94°C, 1 min at 55°C and 1 min at 72°C) and a termination cycle (1 min at 94°C, 2 min at 55°C and 3 min at 72°C).

One per cent of the outer PCR product was used as the template DNA for the second round inner PCR. The conditions of inner PCR were the same except the annealing temperature was 57°C.

The inner PCR products were purified by phenol–chloroform extraction and ethanol precipitation, digested with restriction endonuclease *Bst*NI or *Hph*I, separated by 12% polyacrylamide gel electrophoresis and visualized by ethidium bromide staining (Figure 2).

In each PCR, the DNA of the SW480 colon carcinoma cell line, which is hemizygous for a mutation in K-*ras* codon 12 (Capon et al, 1983), and the DNA of the HCT116 colon carcinoma cell line, which contains a K-*ras* codon 13 aspartate mutation (Jiang et al, 1989), were used as templates for positive controls. Sterilized water was used instead of template DNA and run in parallel in

*© Cancer Research Campaign 1997*

**Table 2** K-*ras* gene mutations in 11 benign adenomas with various grades of dysplasia

| **K-*ras* gene mutation**[a] | **No. of cases** |
|---|---|
| Heterogeneity evident | |
| mild(–) moderate(+) | 1 |
| mild(–) moderate(–) moderate*(+) | 2 |
| moderate(–) moderate*(+) | 1 |
| Heterogeneity not apparent | |
| mild(–) moderate(–) | 5 |
| mild(+) moderate(+) | 2 |

[a] K-*ras* gene mutation, status of K-*ras* gene mutation in the 11 benign adenomas with areas of various grades of dysplasia: mild, mild dysplasia; moderate, moderate dysplasia; moderate*, apparently more advanced compared with the adjacent region with moderate dysplasia but could not be diagnosed as severe dysplasia; (–), mutation negative; (+), mutation positive.

**Table 3** Status of K-*ras* gene mutation in 36 adenomas with focal carcinoma

| **K-ras gene mutation**[a] | **No. of cases** |
|---|---|
| A(–) C(–) | 15 |
| A(+) C(+) | 10 |
| A(–) C(+) | 4 |
| A(+) C(–) | 7 |

[a]K-*ras* gene mutation, status of K-*ras* gene in focal carcinoma and adjacent adenoma in the same case: A, adenoma; C, focal carcinoma; (–) mutation negative; (+) mutation positive.

**Table 4** Type of K-*ras* gene mutation in ten colorectal adenomas with focal carcinoma

| **Case no.** | **Codon** | **Type of mutation** |
|---|---|---|
| 7A | 13 | GAC |
| C | 13 | GAC |
| 27A | 13 | GAC |
| C | 13 | GAC |
| 30A | 12 | ND |
| C | 13 | ND |
| 39A | 12 | GAT |
| C | 12 | GTT |
| 40A | 12 | GAT |
| C | 12 | GAT |
| 41A | 12 | GAT |
| C | 12 | GAT |
| 48A | 12 | GAT |
| C | 12 | GAT |
| 51A | 12 | AGT |
| C | 12 | AGT |
| 52A | 12 | GTT |
| C | 12 | GTT |
| 57A | 12 | NA |
| C | 12 | NA |

NA, not available; ND, not done; A, adenoma; C, focal carcinoma.

agarose gels in each PCR as the negative control. PCR, enzyme digestion and electrophoresis proceeded at least twice to confirm the reproducibility of the results.

**Figure 3** Sequencing of the inner PCR products. (**A**) Case 39 exhibits a G-to-A base change in the adenoma and a G-to-T base change in the focal carcinoma at the second position of K-*ras* codon 12. (**B**) Case 40 exhibits a G-to-A base change at the second position of K-*ras* codon 12 in both the adenoma and the focal carcinoma

### Sequencing of PCR products

Some of the K-*ras* gene mutations in adenomas with focal carcinoma were determined by direct sequencing using a Takara *Taq* cycle sequencing kit (Takara Shuzo, Kyoto, Japan), according to the manufacturer's protocol.

### RESULTS

### Frequency of K-*ras* gene mutation in each grade of dysplasia and in focal carcinomas

We scraped out 36 regions of mild dysplasia, 49 of moderate dysplasia and 36 of focal carcinoma from 32 benign adenomas and 36 adenomas with focal carcinoma, as shown in Table 1. The frequencies of K-*ras* gene mutation in mild dysplasia, moderate dysplasia and focal carcinoma were 19% (7/36), 51% (25/49) and 39% (14/36) respectively. There was a statistically significant difference in the frequency of K-*ras* mutations between the regions of mild and moderate dysplasia ($P = 0.034$, Fisher's exact test).

© Cancer Research Campaign 1997

### K-*ras* gene mutations in 11 benign adenomas with different grades of dysplasia

Several regions were scraped out from 11 benign adenomas that contained at least two areas with different grades of dysplasia (Table 1C and Table 2). Among these adenomas, four showed heterogeneity of the K-*ras* gene mutations and these were found only in the regions with more advanced dysplasia. Case 90 contained a mutation in the region of moderate, but not mild dysplasia, and three others (cases 89, 91 and 96) had a mutation in the regions with apparently more advanced dysplasia compared with the adjacent regions of moderate dysplasia (represented as moderate*). Heterogeneity of the K-*ras* gene was not evident in the seven other benign adenomas.

### Relationship between malignant conversion and K-*ras* gene mutation

We divided the 36 adenomas with focal carcinoma, listed in Table 1D, into four groups according to the K-*ras* gene status in the adenoma regions and the focal carcinoma regions (Table 3). Seven had a mutation only in the adenoma region and four harboured a mutation only in the focal carcinoma. Ten had a mutation in both the adenoma region and the focal carcinoma region. However, in case 30, the mutation in the adenoma region was detected in codon 12 and that in the focal carcinoma region was in codon 13 (Figure 2). We then determined the type of K-*ras* mutation in the remaining nine cases by sequencing (Figure 3, Table 4). All of the point mutations detected by sequencing were found in the same codon by means of PCR–RFLP. Seven cases had the same type of mutation in both the adenoma and carcinoma regions, but case 39 had a different type (case 57 could not be sequenced owing to limitations in the amount of the DNA samples.). We could not detect K-*ras* gene mutations in either adenoma regions or focal carcinomas in the remaining 15 cases.

Finally, the K-*ras* gene mutations were heterogeneous in 13 of 36 adenomas with focal carcinoma.

### DISCUSSION

Several investigators have reported that the frequency of K-*ras* gene mutations in colorectal adenoma is related to the grade of dysplasia (Vogelstein et al, 1988; Miyaki et al, 1990; Boughdady et al, 1992; Ichii et al, 1993; Soh et al, 1993). Also in this study, the frequency of K-*ras* mutation was obviously higher in the regions with moderate dysplasia than in the regions with mild dysplasia. Our methods, including microscraping of tissue samples from histologically pure subpopulations with different grades of dysplasia should provide more precise information about the contribution of K-*ras* gene mutations to the progression of colorectal tumours: from mild to moderate dysplasia and from adenoma to carcinoma.

Four of the 11 benign adenomas with various regions of different dysplasia exhibited heterogeneous K-*ras* gene mutations. Furthermore, mutations were found only in the regions with more advanced dysplasia (Table 2). In addition, no adverse conditions, such as mild(+) moderate(–), were found in this study. This suggests that adenoma cells with K-*ras* gene mutations form a novel clonal expansion, which always appeared with more advanced dysplasia in this study. Therefore, we speculate that K-*ras* gene mutations contribute to a more advanced morphological phenotype during the late stages of adenoma progression. However, several reports of a high incidence of K-*ras* gene mutations in aberrant crypt foci, which are histologically not dysplastic but hyperplastic, suggest that a K-*ras* gene mutation alone cannot confer dysplastic change (Pretlow et al, 1993; Smith et al, 1994). To clarify whether or not the non-neoplastic epithelial cells with a *ras* mutation develop into adenoma, further cell biological studies are required.

With regard to the relationship between the *ras* gene and malignant conversion, several investigators have claimed that *ras* gene mutations do not contribute to the malignant transformation from adenoma to carcinoma, because the frequency of K-*ras* gene mutation is similar in adenomas with advanced dysplasia and in carcinomas of the colorectum (Vogelstein et al, 1988; Boughdady et al, 1992; Ichii et al, 1993; Soh et al, 1993). The microanalysis in this study enabled us to compare the mutational status of the *ras* gene in the focal carcinoma with that in the adjacent background adenoma in individual cases, thus providing some direct information about the role of K-*ras* gene mutations in malignant transformation. Seven cases had the same type of K-*ras* gene mutation in the adenoma and focal carcinoma (Table 4). A K-*ras* gene mutation, which had already occurred in the adenoma region, might simply have been inherited by the focal carcinoma in these cases. Seven cases had K-*ras* mutations in the adenoma, but not in the focal carcinoma regions [represented as A(+) C(–)], and the focal carcinomas in these cases must have developed from an adenoma cell without K-*ras* mutations although there was another subpopulation with a K-*ras* mutation in the same case. These findings showed that a subpopulation of an adenoma with a K-*ras* gene mutation is not always the most progressive clone that will form a focal carcinoma. Among these examples of A(+) C(–), case 26 and OC4 contained regions with mild dysplasia, moderate dysplasia and focal carcinoma (Table 1D), and K-*ras* gene mutations were found only in the region with moderate dysplasia. Although K-*ras* gene mutations could confer the progression from mild to moderate dysplasia, focal carcinomas were derived from regions without K-*ras* mutations in these cases. In four cases with A(–) C(+), K-*ras* gene mutations might make some contribution to the malignant transformation. Overall, we concluded that K-*ras* gene mutations do not contribute directly to the malignant transformation from adenoma to carcinoma. In this respect, *p53* gene alteration is the most probable candidate contributing to the malignant conversion, as we reported (Ohue et al, 1994).

Finally, the K-*ras* gene was heterogeneous in four of the 11 benign adenomas with various regions of different grades of dysplasia and in 13 of the 36 adenomas with focal carcinoma. Although the precise frequency remains unclear, heterogeneity of the K-*ras* gene is a very common event in colorectal adenomas. K-*ras* gene mutations might not be the initial event, but rather occur during adenoma progression resulting in the heterogeneous status. As reported previously, *APC* mutations already occur in very small adenomas and can be the initiating alteration in the colorectal adenoma formation (Powell et al, 1992). K-*ras* gene heterogeneity in colorectal adenoma has also been shown in four of seven colorectal adenomas (Shibata et al, 1993). The present findings are in line with their report, although we increased the number of samples and the variety to include focal carcinomas.

Furthermore, the microanalysis of colorectal adenomas with different grades of dysplasia used in this study is thought to be a very useful means of clarifying the genetic alteration involved in colorectal tumorigenesis.

© Cancer Research Campaign 1997

### ACKNOWLEDGEMENTS

We are grateful to Ms Kyoko Tamura for her technical assistance in preparation of tissue sections. This work was supported in part by Grants-in-Aid for Scientific Research from the Ministry of Education and the Ministry of Health and Welfare, Science and Culture of Japan, and a Grant from the Research Fund for Digestive Molecular Biology.

### REFERENCES

Baker SJ, Fearon ER, Nigro JM, Hamilton SR, Preisinger AC, Jessup JM, Vantuinen P, Ledbetter DH, Barker DF, Nakamura Y, White R and Vogelstein B (1989) Chromosome 17 deletions and *p53* gene mutations in colorectal carcinomas. *Science* **244**: 217–221

Bell SM, Scott N, Cross D, Sagar P, Lewis FA, Blair GE, Taylor GR, Dixon MF and Quirk P (1993) Prognostic value of *p53* overexpression and c-Ki-*ras* gene mutations in colorectal cancer. *Gastroenterology* **104**: 57–64

Bos JL, Fearon ER, Hamilton SR, Verlaan-DE-Vries M, Van-Boom JH, Van-Der-EB AJ and Vogelstein B. (1987). Prevalence of *ras* gene mutations in human colorectal cancers. *Nature* **327**: 293–297

Boughdady IS, Kinsella AR, Haboubi NY and Schofield PF. (1992). *K-ras* gene mutations in adenomas and carcinomas of the colon. *Surg Oncol* **1**: 275–282

Burmer GC and Loeb LA. (1989). Mutations in the *KRAS2* oncogene during progressive stages of human colon carcinoma. *Proc Natl Acad Sci USA* **86**: 2403–2407

Capon DJ, Seeburg PH, McGrath JP, Hayflick JS, Edman U, Levinson Ad and Goeddel DV. (1983). Activation of Ki-*ras2* gene in human colon and lung carcinomas by two different point mutations. *Nature* **304**: 507–513

Fearon ER. (1993). K-*ras* gene mutation as a pathogenetic and diagnostic marker in human cancer. *J Natl Cancer Inst* **85**: 1978–1980

Fearon ER, CHO KR, Nigro JM, Kern SE, Simons JW, Ruppert JM, Hamilton SR, Preisinger AC, Thomas G, Kinzler KW and Vogelstein B. (1990). Identification of a chromosome 18q gene that is altered in colorectal cancers. *Science* **247**: 49–56

Forrester K, Almoguera C, Han K, Grizzle WE and Perucho M. (1987). Detection of high incidence of K-*ras* oncogenes during human colon tumorigenesis. *Nature* **327**: 298–303

Groden J, Thliveris A, Samowitz W, Carlson M, Gelbert L, Albertsen H, Joslyn G, Stevens J, Spirio L, Robertson M, Sargeant L, Krapcho K, Wolff E, Burt R, Hughes JP, Warrington J, Mcpherson J, Wasmuth J, Paslier DL, Abderrahim H, Cohen D, Leppert M and White R. (1991). Identification and characterization of the familial adenomatous polyposis coli gene. *Cell* **66**: 589–600

Hinds PW, Finlay CA, Quartin RS, Baker SJ, Fearon ER, Vogelstein B and Levine AJ. (1990). Mutant *p53* DNA clones from human colon carcinomas cooperate with *ras* in transforming primary rat cells: a comparison of the 'hot spot' mutant phenotypes. *Cell Growth Different* **1**: 571–580

Ichii S, Takeda S, Horii A, Nakatsuru S, Miyoshi Y, Emi M, Fujiwara Y, Koyama K, Furuyama J, Utsunomiya J and Nakamura Y. (1993). Detailed analysis of genetic alterations in colorectal tumors from patients with and without familial adenomatous polyposis (FAP). *Oncogene* **8**: 2399–2405

Jiang W, Kahn SM, Guillem JG, L, SH and Weinstein IB. (1989). Rapid detection of *ras* oncogenes in human tumors: applications to colon, esophageal, and gastric cancer. *Oncogene* **4**: 923–928

Kawasaki Y, Monden T, Morimoto H, Murotani M, Miyoshi Y, Kobayashi T, Shimano T and Mori T. (1992). Immunohistochemical study of *p53* expression in microwave-fixed, paraffin-embedded sections of colorectal carcinoma and adenoma. *Am J Clin Pathol* **97**: 244–249

Kinzler KW, Nilbert MC, SU LK, Vogelstein B, Bryan TM, Levy DB, Smith KJ, Preisinger AC, Hedge P, Mckechnie D, Finniear R, Markham A, Groffin J, Boguski MS, Altschul SF, Horii A, Ando H, Miyoshi Y, Miki Y, Nishisho I and Nakamura Y. (1991). Identification of FAP locus genes from chromosome 5q21. *Science* **253**: 661–665

Kinzler KW, Nilbert MC, Vogelstein B, Bryan TM, Levy DB, Smith KJ, Preisinger AC, Hamilton SR, Hedge P, Markham A, Carlson M, Joslyn G, Groden J, White R, Miki Y, Miyoshi Y, Nishisho I and Nakamura Y. (1991). Identification of a gene located at chromosome 5q21 that is mutated in colorectal cancers. *Science* **251**: 1366–1370

McGrath JP, Capon DJ, Smith DH, Chen EY, Seeburg PH, Goeddel DV and Levinson AD. (1983). Structure and organization of the human Ki-*ras* proto-oncogene and a related processed pseudogene. *Nature* **304**: 501–506

Miyaki M, Seki M, Okamoto M, Yamanaka A, Maeda Y, Tanaka K, Kikuchi R, Iwama T, Ikeuchi T, Tonomura A, Nakamura Y, White R, Miki Y, Utsunomiya J and Koike M (1990) Genetic changes and histopathological types in colorectal tumors from patients with familial adenomatous polyposis. *Cancer Res* **50:** 7166–7173

Nishisho I, Nakamura Y, Miyoshi Y, Miki Y, Ando H, Horii A, Koyama K, Utsunomiya J, Baba S and Hedge P (1991) Mutations of chromosomes 5q21 genes in FAP and colorectal cancer patients. *Science* **253:** 665–669

Ohue M, Tomita N, Monden T, Fujita M, Fukunaga M, Takami K, Yana I, Ohnishi T, Enomoto T, Inoue M, Shimano T and Mori T (1994) A frequent alteration of *p53* gene in carcinoma in adenoma of colon. *Cancer Res* **54:** 4798–4804

Powell SM, Zilz N, Beazer-Barclay Y, Bryan TM, Hamilton SR, Thibodeau SN, Vogelstein B and Kinzler KW (1992) *APC* mutations occur early during colorectal tumorigenesis. *Nature* **359:** 235–237

Pretlow TP, Brasitus TA, Fulton NC, Cheyer C and Kaplan EL (1993) *K-ras* mutations in putative preneoplastic lesions in human colon. *J Natl Cancer Inst* **85:** 2004–2007

Sambrook JEF and Maniatis T (1989) *Molecular Cloning: A Laboratory Manual*, 2nd edn. Cold Spring Harbor Laboratory Press: Cold Spring Harbor, NY

Shaw P, Tardy S, Benito E, Obrador A and Costa J (1991) Occurrence of *Ki-ras* and *p53* mutations in primary colorectal tumors. *Oncogene* **6:** 2121–2128

Shibata D, Schaeffer J, Li ZH, Capella G and Perucho M (1993) Genetic heterogeneity of the c-K-*ras* locus in colorectal adenomas but not in adenocarcinomas. *J Natl Cancer Inst* **85:** 1058–1063

Shirasawa S, Furuse M, Yokoyama N and Sasazuki T. (1993). Altered growth of human colon cancer cell lines disrupted at activated Ki-*ras*. *Science* **260**: 85–88

Smith AJ, Stern HS, Penner M, Hay K, Mitri A, Bapat BV and Gallinger S. (1994). Somatic *APC* and K-*ras* codon 12 mutations in aberrant crypt foci from human colons. *Cancer Res* **54**: 5527–5530

Soh K, Yanagisawa A, Hiratsuka H, Sugano H and Kato Y. (1993). Variation in K-*ras* codon 12 point mutation rate with histological atypia within individual colorectal tumors. *Jpn J Cancer Res* **84**: 388–393

Vogelstein B, Fearon ER, Hamilton SR, Kern SE, Preisinger AC, Leppert M, Nakamura Y, White R, Smits AM and Bos JL (1988) Genetic alterations during colorectal tumor development. *N Engl J Med* **319**: 525–532

© Cancer Research Campaign 1997